# Membrane Remodeling by a Bacterial Phospholipid-Methylating Enzyme

**DOI:** 10.1128/mBio.02082-16

**Published:** 2017-02-14

**Authors:** Linna Danne, Meriyem Aktas, Andreas Unger, Wolfgang A. Linke, Ralf Erdmann, Franz Narberhaus

**Affiliations:** a Microbial Biology, Faculty of Biology, Ruhr University Bochum, Bochum, Germany; b Institute of Biochemistry and Pathobiochemistry, Faculty of Medicine, Ruhr University Bochum, Bochum, Germany; c Department of Cardiovascular Physiology, Faculty of Medicine, Ruhr University Bochum, Bochum, Germany; Karlsruhe Institute of Technology (KIT)

## Abstract

Membrane deformation by proteins is a universal phenomenon that has been studied extensively in eukaryotes but much less in prokaryotes. In this study, we discovered a membrane-deforming activity of the phospholipid *N*-methyltransferase PmtA from the plant-pathogenic bacterium *Agrobacterium tumefaciens*. PmtA catalyzes the successive three-step *N*-methylation of phosphatidylethanolamine to phosphatidylcholine. Here, we defined the lipid and protein requirements for the membrane-remodeling activity of PmtA by a combination of transmission electron microscopy and liposome interaction studies. Dependent on the lipid composition, PmtA changes the shape of spherical liposomes either into filaments or small vesicles. Upon overproduction of PmtA in *A. tumefaciens*, vesicle-like structures occur in the cytoplasm, dependent on the presence of the anionic lipid cardiolipin. The N-terminal lipid-binding α-helix (αA) is involved in membrane deformation by PmtA. Two functionally distinct and spatially separated regions in αA can be distinguished. Anionic interactions by positively charged amino acids on one face of the helix are responsible for membrane recruitment of the enzyme. The opposite hydrophobic face of the helix is required for membrane remodeling, presumably by shallow insertion into the lipid bilayer.

## IMPORTANCE

The ability to alter the morphology of biological membranes is known for a small number of some bacterial proteins. Our study adds the phospholipid *N*-methyltransferase PmtA as a new member to the category of bacterial membrane-remodeling proteins. A combination of *in vivo* and *in vitro* methods reveals the molecular requirements for membrane deformation at the protein and phospholipid level. The dual functionality of PmtA suggests a contribution of membrane biosynthesis enzymes to the complex morphology of bacterial membranes.

## INTRODUCTION

Protein-mediated membrane remodeling is fundamental for numerous biological processes in all domains of life (1–3). In eukaryotes, membrane fusion and fission processes play central roles in a broad spectrum of cellular events, including exocytosis, endocytosis, cytokinesis, and intracellular membrane trafficking (2–5). Remodeling of membranes by fission or fusion has been well studied in eukaryotes, and a number of dedicated proteins have been identified; these proteins are required for membrane shaping, curvature enhancement, and vesicle formation (4, 6–9). In comparison, the number of proteins known to be directly responsible for such events in bacteria is low (10). Membrane remodeling in bacteria occurs at both the cytoplasmic membrane and the outer membrane (in the case of Gram-negative organisms). Dynamic membrane shaping mainly requires the active involvement of curvature-inducing proteins. A number of such proteins have been described and characterized. For example, bacterial members of the dynamin family were shown to have membrane-shaping activity. Proteins of the dynamin superfamily mediate restructuring of the membrane by polymerizing upon lipid bilayers and forcing regions of high curvature (8, 10). The chemical energy for membrane remodeling by dynamins depends on nucleotide turnover. The mechanisms of helix assembly, GTP hydrolysis, and generation of lipid curvature were precisely investigated for the bacterial dynamin-like protein (BDLP) from the cyanobacterium *Nostoc punctiforme* (11). In addition, bacterial cell division proteins like MinE from *Escherichia coli* have been shown to alter the morphology of liposomes *in vitro* and self-assemble into fibrillar structures on lipid bilayers (12, 13). The fission protein B (FisB) from *Bacillus subtilis* was found to be directly responsible for membrane remodeling during the developmental process of spore formation (14). Further membrane restructuring events in bacteria were observed upon overexpression of a lipid glycosyltransferase (GT) from *Acholeplasma laidlawii*, which causes massive formation of intracellular membrane vesicles (IMVs) when expressed in *E. coli* (15, 16). Similarly, the *E. coli* GTs, MurG and LpxB, which are involved in peptidoglycan and lipopolysaccharide biosynthesis, respectively, induce the formation of IMVs (17). Another bacterial curvature-inducing protein is the actin homolog MreB from *Thermotoga maritima*, which interacts with the membrane via an N-terminal amphipathic helix and assembles into double filaments on the lipid bilayer, which can induce local membrane curvature (18). Via electron tomography, it was shown that the MreB homolog MamK from *Magnetospirillum magneticuma* supports membrane invagination of magnetosomes, which are unique organelles of magnetotactic bacteria that contain magnetic iron minerals (19, 20).

Here, we report that the phospholipid *N*-methyltransferase PmtA from the plant pathogen *Agrobacterium tumefaciens* has the capacity to deform lipid membranes. PmtA is involved in the biogenesis of *A. tumefaciens* membranes and synthesizes the membrane lipid phosphatidylcholine (PC). A PC-deficient *A. tumefaciens* strain lacks the type IV secretion system, which is essential for T-DNA transfer and tumor formation (21). PmtA catalyzes the successive *N*-methylation of phosphatidylethanolamine (PE) via the intermediates monomethyl-phosphatidylethanolamine (MMPE) and dimethyl-phosphatidylethanolamine (DMPE) to PC and uses *S*-adenosylmethionine (SAM) as the methyl donor (22). The overall enzyme activity is stimulated by negatively charged lipids (22, 23). SAM binding occurs via a conserved SAM-binding motif localized at the N terminus of the protein (24). PmtA binds SAM only in the presence of its substrates, PE, MMPE, and DMPE, or the end product PC (22). In order to be active, PmtA needs to be recruited to the cytoplasmic membrane where the substrate lipids are available. Membrane association of PmtA is based on electrostatic interactions with acidic phospholipids and seems to be further stabilized by hydrophobic insertion into the core of the lipid bilayer (23). Of the two alpha-helical lipid-binding regions (αA and αF), the N‑terminal αA region is predominantly required for membrane binding. Membrane attachment to anionic lipids is dependent on the membrane-binding motif constituted of basic (R8 and K12) and hydrophobic (F19) residues (23).

In the present study, we provide evidence for a membrane-remodeling capacity of PmtA, which is strictly dependent on the presence of the anionic membrane lipid cardiolipin (CL). The bifunctional αA region, which confers membrane binding by one face of the helix, is also responsible for membrane remodeling by the other face of the helix.

## RESULTS

### PmtA remodels liposomes.

**FIG 1:**
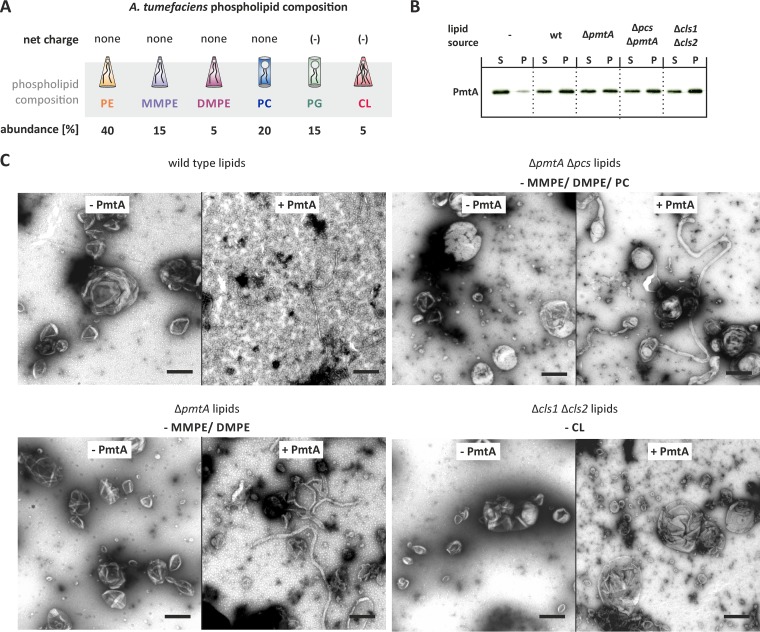
PmtA deforms model membranes *in vitro*. (A) Phospholipid composition of *A. tumefaciens* membranes (25). (B) Liposome cosedimentation assay with liposomes containing lipids of different *A. tumefaciens* strains. Recombinant PmtA (10 µM) was incubated with ~0.5 mg/ml liposomes. Supernatant (S) and pellet (P) fractions were analyzed by SDS-PAGE and Western blotting. PmtA was detected with an anti-His antibody. wt, wild type. (C) Membrane-remodeling activity of PmtA assayed via the liposome-remodeling assay. Liposomes (~0.5 mg/ml) containing the indicated lipids from different *A. tumefaciens* strains were incubated with 10 µM recombinant PmtA (+ PmtA). Samples were incubated for 30 min at room temperature. Liposomes were negatively stained using 1% uranyl acetate and imaged with a Philips 420 transmission microscope. Data are representative of three independent experiments. Bars represent 100 nm. Abbreviations: PE, phosphatidylethanolamine; MMPE, monomethyl-phosphatidylethanolamine; DMPE, dimethyl-phosphatidylethanolamine; PC, phosphatidylcholine; PG, phosphatidylglycerol; CL, cardiolipin.

PmtA is a peripheral membrane protein transiently binding to the cytoplasmic leaflet of the inner membrane. Previously we demonstrated via *in vitro* lipid-binding studies that the association with liposomes is strictly dependent on the presence of anionic lipids (23). It is known that certain peripheral membrane proteins induce membrane curvature causing membrane bending (2–4). In this study, we aimed at analyzing the consequences of lipid binding by PmtA for the morphology of liposomes and examined the morphology of liposomes in the absence and presence of recombinant PmtA via transmission electron microscopy (TEM). To establish liposome-remodeling assays for PmtA, we tested different salt concentrations and protein-to-lipid ratios in pilot experiments and found optimal conditions with KH_2_PO_4_ buffer (50 mM; pH 8.0) and a protein-to-lipid ratio of 1:75. To evaluate the role of the phospholipid composition in membrane deformation, we used liposomes containing lipids extracted from different *A. tumefaciens* strains. The most abundant phospholipid in *A. tumefaciens* membranes is PE (Fig. 1A) (25). Further, *A. tumefaciens* produces the methylated PE derivatives MMPE, DMPE, and PC and the anionic phospholipids phosphatidylglycerol (PG) and CL (26, 27). The phosphate-free ornithine lipids OLS1 and OLS2 (28) are not depicted in Fig. 1A. Efficient binding of PmtA to liposomes from all four different *A. tumefaciens* strains was confirmed via liposome cosedimentation assays (Fig. 1B). Liposomes containing wild-type *A. tumefaciens* lipids were round with an average size of 200 to 300 nm in the absence of PmtA (Fig. 1C). After incubation with PmtA, they were converted into small vesicles with a diameter of 10 to 20 nm, demonstrating that PmtA is able to remodel liposomes. Lipids from different *A. tumefaciens* phospholipid biosynthesis mutants (21) allowed us to distinguish the roles of individual lipids on membrane deformation by PmtA. Liposomes from an *A. tumefaciens* Δ*pmtA* Δ*pcs* strain (lacking MMPE, DMPE, and PC) were transformed into long cylindrical tubular structures with a diameter of about 20 nm (Fig. 1C), suggesting that PmtA is able to shape liposomes either into small vesicles or tubular filaments. Further, we used liposomes from a Δ*pmtA* strain, which lacks MMPE and DMPE, but still produces PC via the PC synthase pathway, in which PC is synthesized via condensation of choline and CDP-diacylglycerol (29). Liposomes from the Δ*pmtA* strain were converted into long tubular structures by PmtA (Fig. 1C), suggesting that either MMPE or DMPE alone or both intermediates of PC biosynthesis are responsible for membrane vesiculation by PmtA.

To lend further support to the hypothesis that MMPE or DMPE promotes membrane vesiculation by PmtA, we used artificial model membranes with defined phospholipid composition. Preparations containing PE, PG, and CL were supplemented either with MMPE, DMPE, or PC. Binding of PmtA to all liposomes was comparable (see Fig. S1A in the supplemental material). In the absence of PmtA, the liposomes were spherical (Fig. S2A). Consistent with liposomes from the *A. tumefaciens* Δ*pmtA* Δ*pcs* strain, round model membranes containing PE, PG, and CL were tubulated by PmtA into long lipid filaments (Fig. S1B). The presence of MMPE shifted remodeling toward the conversion of small vesicles (Fig. S1C). Similar vesiculation was observed in the presence of DMPE (Fig. S1D). Since PC addition did not alter membrane tubulation activity of PmtA (Fig. S1E), we conclude that MMPE and DMPE are crucial for membrane vesiculation by PmtA.

10.1128/mBio.02082-16.1 FIG S1  Influence of MMPE, DMPE, and PC on the membrane-deforming activity of PmtA. (A) Liposome cosedimentation assay of PmtA with model membranes (PE/PG/CL 7.5:2:0.5) supplemented with either MMPE (PE/PG/CL/MMPE 6:2:0.5:1.5), DMPE (PE/PG/CL/DMPE 6:2:0.5:1.5) or PC (PE/PG/CL/PC 6:2:0.5:1.5). PmtA (10 μM) was incubated with 0.75 mM liposomes. (B to E) Liposome-remodeling assay with model membrane containing MMPE, DMPE, or PC analyzed via TEM. Liposomes (0.75 mM) with the indicated lipid compositions were incubated with 10 µM recombinant PmtA for 30 min at room temperature. Liposomes were negatively stained using 1% uranyl acetate and imaged with a Philips 420 TEM. The bars represent 200 nm. Data are representative of three independent experiments. S, supernatant; P, pellet; PE, phosphatidylethanolamine; MMPE, monomethyl-phosphatidylethanolamine; DMPE, dimethyl-phosphatidylethanolamine; PC, phosphatidylcholine; PG, phosphatidylglycerol; CL, cardiolipin. Download FIG S1, JPG file, 2.9 MB. Copyright © 2017 Danne et al. 2017 Danne et al. This content is distributed under the terms of the Creative Commons Attribution 4.0 International license.

10.1128/mBio.02082-16.2 FIG S2  TEM micrographs of liposomes without incubation with PmtA. The control experiments were performed as described in the legend to Fig. S1. Lipid composition is indicated in the figure. PE, phosphatidylethanolamine; MMPE, monomethyl-phosphatidylethanolamine; DMPE, dimethyl-phosphatidylethanolamine; PC, phosphatidylcholine; PG, phosphatidylglycerol; CL, cardiolipin; PA, phosphatidic acid; PI, phosphatidylinositol, PIP(4), phosphatidylinositol-4-phosphate. Download FIG S2, JPG file, 0.5 MB. Copyright © 2017 Danne et al. 2017 Danne et al. This content is distributed under the terms of the Creative Commons Attribution 4.0 International license.

### Cardiolipin promotes liposome deformation by PmtA.

**FIG 2:**
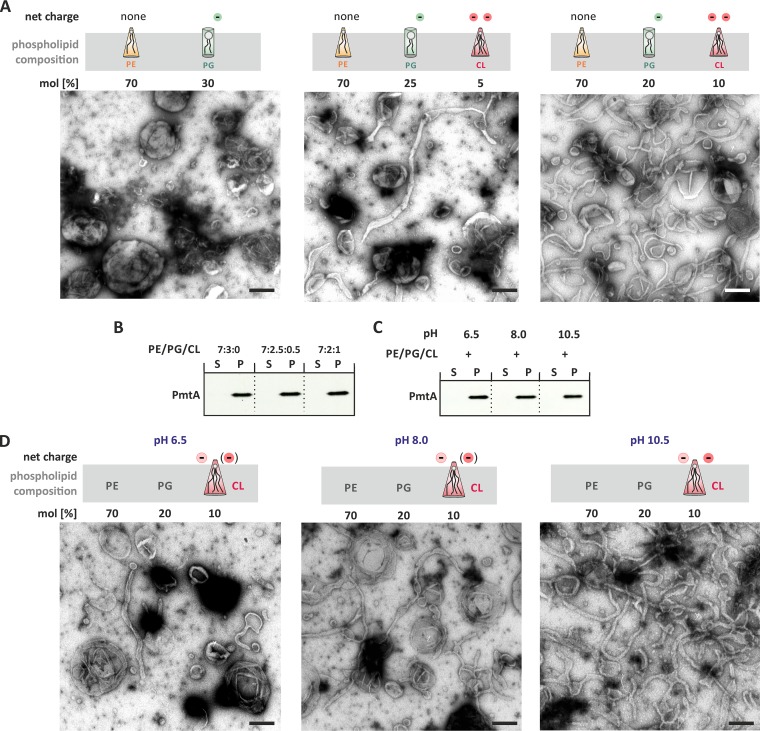
Cardiolipin (CL) is essential for the membrane-remodeling activity of PmtA. (A) Membrane deformation activity of PmtA with liposomes containing increasing CL concentrations analyzed via TEM. The experimental procedure was performed as described in the legend to Fig. 1. (B) Binding affinity of PmtA toward model membranes containing increasing CL concentrations was determined via the liposome cosedimentation assay. (C) Liposome cosedimentation assay of PmtA with model membranes containing PE, PG, and CL at different pHs. (D) Influence of pH on the membrane deformation activity of PmtA investigated via the liposome-remodeling assay. Samples were prepared as described above. All samples contained 10 µM recombinant PmtA and 0.75 mM liposomes. The charge of CL at pH 6.5, 8.0, and 10.5 is indicated. Please note that the proton dissociation behavior of its phosphate groups is a matter of controversy (as depicted in parentheses) (34–36). Data are representative of three independent experiments. Bars represent 100 nm.

As CL is involved in membrane interaction of PmtA (23), we assayed deformation of liposomes containing lipids from the CL-deficient *A. tumefaciens* Δ*cls1* Δ*cls2* strain (30). Liposomes from this strain were hardly deformed by PmtA (Fig. 1C), suggesting that CL is crucial for membrane remodeling by PmtA. To validate this hypothesis, we prepared artificial model membranes with increasing CL concentrations (Fig. 2A). Liposome cosedimentation assays demonstrated that PmtA bound to all three prepared liposome types equally well (Fig. 2B). Despite binding to the membrane, PmtA did not remodel liposomes lacking CL (Fig. 2A). Increasing CL concentrations stimulated membrane tubulation, suggesting that membrane shaping by PmtA is initiated by the presence of the anionic lipid CL.

Further, we investigated the molecular basis for the CL-dependent stimulation of membrane remodeling by PmtA. CL is distinguished from other common membrane glycerolipids by a head group alcohol that is shared by two phosphatidate moieties (31, 32). Hence, under physiological conditions, CL contains two negatively charged phosphate groups. The four acyl chains in CLs cause a small head-to-tail surface ratio compared to other glycerophospholipids, resulting in a conical shape. Due to this geometry, CL tends to form nonlamellar structures (33). We aimed to analyze in detail which property of CL is crucial for membrane remodeling by PmtA. To investigate whether the negative charge of CL is important for the membrane-shaping activity of PmtA, we assayed membrane remodeling at different pH values, assuming that in contrast to PE and PG, the negative charge of CL is influenced by pH changes from 6.5 to 10.5. The proton dissociation behavior of the CL head group is controversial and has been discussed (34–36). On the one hand, in some publications, it is stated that the pK_a_ values of the two phosphate moieties differ by several units and that the head group exists as a monoanion at physiological pH (36). On the other hand, some recent publications showed that the two phosphates have pK_a_ values of about 2.8 and 3.8, resulting in two negative charges at physiological pH (34, 35). Although binding of PmtA to model membranes was similar under all tested conditions (Fig. 2C), membrane deformation was pH responsive (Fig. 2D). Small amounts of lipid tubes were observed at pH 6.5. Membrane tubulation was enhanced at pH 8.0 and strongly increased at pH 10.5 (Fig. 2D). These results might indicate that the membrane-remodeling activity of PmtA correlates with the charge of CL. However, we cannot exclude the possibility that pH-responsive membrane tubulation by PmtA is due to changes in the ionization state of the protein.

To support the assumption that a dual negative charge is sufficient to initiate membrane deformation, we performed the liposome tubulation assay with model membranes containing PE and PG supplemented with phosphatidylinositol 4-phosphate (PI4P), which also possesses two anionic moieties but is cylindrical (Fig. S3). Without PmtA all prepared liposomes were spherical (Fig. S2B). Phosphatidylinositol (PI) was used as a control, and liposomes containing PE, PG, and PI were not deformed by PmtA (Fig. S3). Almost all CL-containing liposomes were tubulated. In contrast, about 10% of the PI4P-containing liposomes were tubulated by PmtA (Fig. S3). These results suggest that the charge of the lipid head group is important for membrane deformation by PmtA but does not yet exclude a role of the shape of the phospholipid. Thus, we further tested whether the conical shape of CL is important for membrane remodeling by PmtA and used model membranes containing the anionic and conical phosphatidic acid (PA). Liposomes from both preparations were spherical in the absence of PmtA (Fig. S2C). Liposomes containing PE, PG, and PA were remodeled to lipid tubes by PmtA but with less efficacy than in the presence of CL (Fig. S4A and B). Binding to PA-containing liposomes was also reduced than binding to CL-containing liposomes (Fig. S4C). Taken together, the combination of the net charge and the conical geometry of CL seems to be responsible for the membrane-remodeling activity of PmtA.

10.1128/mBio.02082-16.3 FIG S3  Membrane-remodeling assay with PI derivatives. Membrane-remodeling activity of PmtA in the presence of model membranes containing different PI species. (A to C) Liposomes contained PE, PG, and CL (A), PE, PG, and PI (B), or PE, PG and PIP(4) (C) with the indicated lipid concentrations. Samples were prepared as described in the legend to Fig. S1. (D) Liposome cosedimentation assay of PmtA with model membranes containing different PI species. Lipid composition of the liposomes used is described above. PmtA (10 µM) was incubated with 0.75 mM liposomes. Fractions of supernatant (S) and pellet (P) were analyzed by SDS-PAGE and Western blot analysis. PmtA was detected with an anti-His antibody. The bars represent 100 nm. Data are representative of three independent experiments. PE, phosphatidylethanolamine; PG, phosphatidylglycerol; CL, cardiolipin; PI, phosphatidylinositol; PIP(4), phosphatidylinositol-4-phosphate. Download FIG S3, JPG file, 0.3 MB. Copyright © 2017 Danne et al. 2017 Danne et al. This content is distributed under the terms of the Creative Commons Attribution 4.0 International license.

10.1128/mBio.02082-16.4 FIG S4  The cone shape of lipids is important to initialize membrane deformation by PmtA. (A and B) Membrane-remodeling activity of PmtA in the presence of model membranes supplemented with CL (A) or PA (B) with the indicated lipid concentrations. Samples were incubated for 30 min at room temperature. The experimental procedure was performed as described in the legend to Fig. S2. (C) Liposome cosedimentation assay of PmtA with model membranes containing CL or PA. The experimental procedure was performed as described in the legend to Fig. S1. The bars represent 100 nm. Data are representative of three independent experiments. S, supernatant; P, pellet; PE, phosphatidylethanolamine; PG, phosphatidylglycerol; CL, cardiolipin; PA, phosphatidic acid. Download FIG S4, JPG file, 0.2 MB. Copyright © 2017 Danne et al. 2017 Danne et al. This content is distributed under the terms of the Creative Commons Attribution 4.0 International license.

Next, we tested whether membrane deformation by PmtA is influenced by membrane fluidity. The fluidity of lipid bilayers is affected by a number of parameters, including saturation of the fatty acids (37). We analyzed membrane-remodeling activity of PmtA with liposomes supplemented with different CL species (C_18:1_ and C_18:2_). Liposomes from both preparations were spherical (Fig. S2D). We found fewer tubulated structures under conditions of more double bonds in CL, creating a less rigid and viscous lipid bilayer (Fig. S5). Thus, membrane remodeling by PmtA is enhanced with increasing membrane fluidity.

10.1128/mBio.02082-16.5 FIG S5  Membrane remodeling by PmtA is enhanced with decreasing CL saturation. (A and B) Membrane-remodeling capacity of PmtA in the presence of model membranes containing CL C_18:1_ (A) or C_18:2_ (B). Liposomes (0.75 mM) containing PE, PG, and CL (7:2:1) were incubated with 10 µM recombinant PmtA. The experimental procedure was performed as described in the legend to Fig. S2. (C) Liposome cosedimentation assay of PmtA with model membranes containing different CL species. PmtA (10 µM) was incubated with 0.75 mM liposomes. Experiments were performed as described in the legend to Fig. S1. The bars represent 100 nm. Download FIG S5, JPG file, 0.2 MB. Copyright © 2017 Danne et al. 2017 Danne et al. This content is distributed under the terms of the Creative Commons Attribution 4.0 International license.

Taken together, our results demonstrate that membrane remodeling by PmtA is strongly affected by the physicochemical properties of the lipid bilayer. Due to its conical shape and net charge, CL is a prerequisite for membrane remodeling by PmtA. The presence of MMPE shifts filament formation by PmtA toward vesicle formation.

### The membrane-binding region αA drives the membrane-remodeling activity of PmtA.

**FIG 3:**
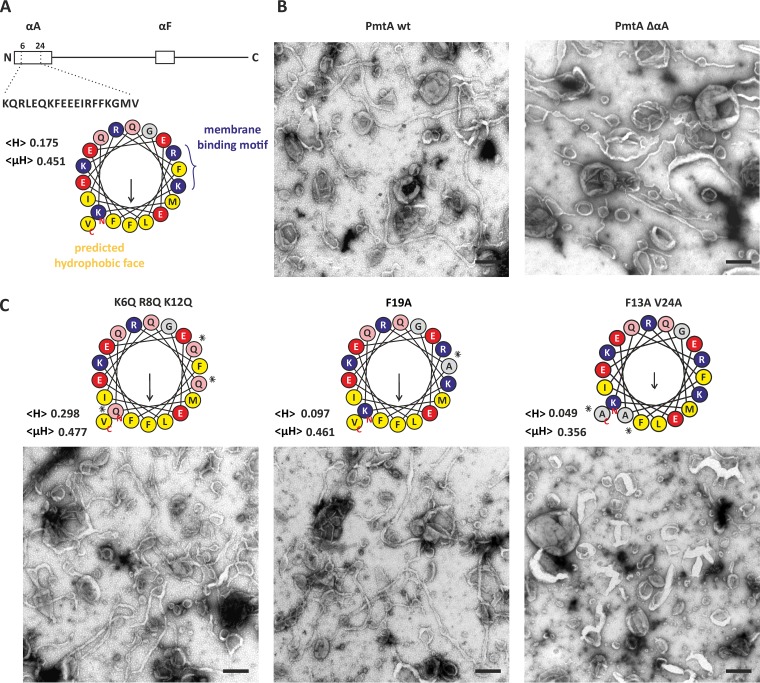
Membrane-remodeling activity of different PmtA variants. (A, top) Schematic representation of PmtA with highlighted membrane-binding regions αA and αF. (Bottom) Helical wheel illustration of αA (amino acids 6 to 24) generated via HELIQUEST (38). The mean hydrophobicity (<H>) and the hydrophobic moment (<µH>) are indicated in the figure. Hydrophobic residues (yellow), basic residues (blue), acidic residues (red), glutamine (rose), and uncharged residues (gray) are indicated. (B and C) Liposome-remodeling assay with truncated PmtA variant (ΔαA) (B) and different point mutants (K6Q R8Q K12Q, F19A, and F13A V24A) (C) with model membranes containing a lipid mixture of PE, PG, and CL (7:2:1) compared to wild-type (wt) PmtA. The experimental procedure was performed as described in the legend to Fig. 1. Data are representative of three independent experiments. Bars represent 100 nm. Asterisks mark exchanged residues.

PmtA contains two membrane-binding regions with the N-terminal αA helix being the predominant binding site (23). According to the HELIQUEST program (38), αA contains an amphiphilic region with a hydrophobic face localized opposite the membrane-binding motif (Fig. 3A). To analyze the role of αA in liposome remodeling by PmtA, we used a truncated PmtA variant (ΔαA) lacking the αA region and analyzed the ability to alter the shape of model membrane containing PE, PG, and CL (Fig. 3B). As shown in Fig. S2E, all liposomes were spherical in the absence of any protein. Compared to wild-type PmtA, ΔαA shows reduced ability to remodel membranes, suggesting that the missing αA helix is important for this activity.

Site-directed mutagenesis was aimed at pinpointing specific residues in αA responsible for membrane remodeling. Mutation of the basic residues K6, R8, and K12 to glutamine or exchange of the hydrophobic residue F19 to alanine in the membrane-binding motif did not affect membrane tubulation by PmtA (Fig. 3C). In contrast, exchange of residues in the predicted hydrophobic face (F13A V24A) abolished the membrane-remodeling activity of PmtA (Fig. 3C). Circular dichroism (CD) spectroscopy verified that the F13A V24A variant was properly folded (Fig. S6). Thus, we concluded that F20 and V24 in the hydrophobic region are required for tubulation activity of PmtA.

10.1128/mBio.02082-16.6 FIG S6  Secondary structure analysis of PmtA variant F13A V24A. Secondary structure of wt PmtA (blue) and F13A V24A PmtA (black) were analyzed via CD spectroscopy. CD spectra of recombinant proteins (5 µM) were recorded 10 times between 190 and 270 nm in 50 mM potassium buffer (pH 8.0) with a Jasco 715 spectropolarimeter at room temperature. Secondary structure prediction was performed via the K2D3 server (57). Download FIG S6, TIF file, 0.6 MB. Copyright © 2017 Danne et al. 2017 Danne et al. This content is distributed under the terms of the Creative Commons Attribution 4.0 International license.

**FIG 4:**
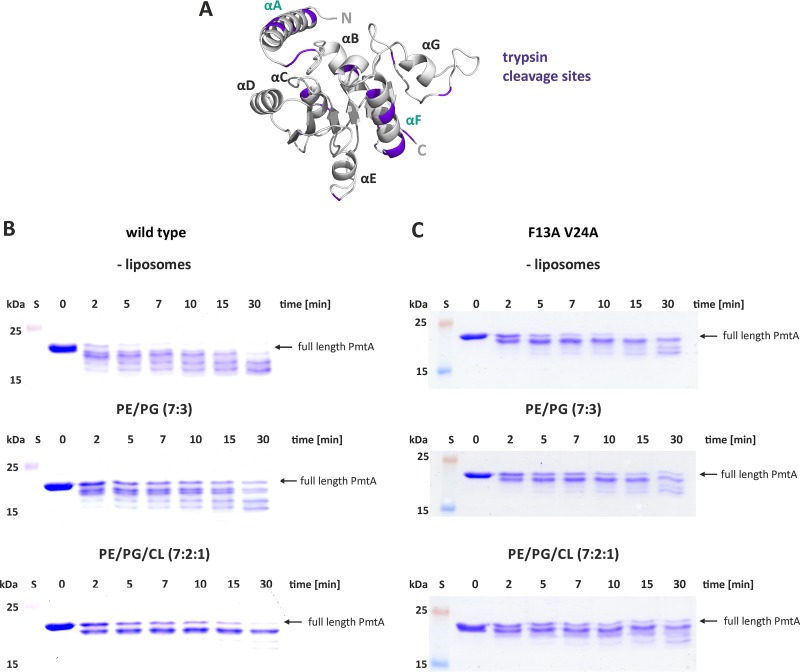
Trypsin protection assay of wild-type PmtA and PmtA with F13A V24A PmtA. (A) Homology model of PmtA generated with I-tasser and visualized with Pymol. Trypsin restriction sites are indicated (violet). (B and C) Protease protection assay with wild-type PmtA (B) and PmtA with F13A V24A (C). Recombinant PmtA (10 µM) was incubated with 0.75 mM liposomes with the indicated lipid composition for 30 min at room temperature. Then 10 µg ml^−1^ trypsin was added, and samples were taken at defined time points as indicated in the figure. Degradation of PmtA was analyzed via SDS-PAGE. Data are representative of three independent experiments. S, standard.

The importance of the hydrophobic face for membrane tubulation suggests an insertion of this region into the membrane core. To confirm this assumption experimentally, we conducted trypsin sensitivity assays in the presence and absence of model membranes containing either PE and PG or PE, PG, and CL. As shown in Fig. 4A, PmtA harbors multiple potential trypsin cleavage sites (basic residues, such as lysines and arginines). In the absence of liposomes, PmtA was readily cleaved into various products after 2 min (Fig. 4B). Incubation of PmtA with liposomes containing PE and PG (7:3) delayed degradation of PmtA by trypsin (Fig. 4B). Most interestingly, the presence of CL almost completely abrogated trypsin digestion and resulted in a single product most likely due to an accessible internal cleavage site in the protein.

PmtA contains an N-terminal polyhistidine tag for protein purification. We did not detect any PmtA products with an anti-His antibody after incubation with trypsin (Fig. S7), suggesting that the N terminus of PmtA is removed by the protease. To support the role of the N-terminal αA region in membrane insertion, we subjected the PmtA variant ΔαA to limited proteolysis. Limited proteolysis of this truncated variant was completely independent of membrane addition (Fig. S8), providing further evidence that the αA region in PmtA is protected by CL-containing membranes.

10.1128/mBio.02082-16.7 FIG S7  PmtA is N terminally degraded by trypsin**.** PmtA (10 µM) was incubated with trypsin (10 µg/ml) for 30 min at room temperature. Samples were taken at defined time points as indicated in the figure. Degradation of PmtA was analyzed via SDS-PAGE and Western blotting. PmtA was detected with an anti-His antibody. Download FIG S7, TIF file, 0.2 MB. Copyright © 2017 Danne et al. 2017 Danne et al. This content is distributed under the terms of the Creative Commons Attribution 4.0 International license.

10.1128/mBio.02082-16.8 FIG S8  PmtA variant ΔαA is not protected against trypsin by the presence of liposomes**.** Protease protection assay of the PmtA ΔαA in the presence and absence of liposomes containing a lipid mixture of PE and PG or PE, PG, and CL. ΔαA (10 µM) was incubated with 0.75 mM liposomes for 30 min at room temperature. Afterward 10 µg/ml trypsin was added, and samples were taken at defined time points as indicated in the figure. Degradation of PmtA was analyzed via SDS-PAGE. Data are representative of three independent experiments. Download FIG S8, JPG file, 0.1 MB. Copyright © 2017 Danne et al. 2017 Danne et al. This content is distributed under the terms of the Creative Commons Attribution 4.0 International license.

Knowing that the hydrophobic face of αA is responsible for membrane remodeling (Fig. 3C), we tested whether the PmtA variant F13A V24A is protected from trypsin digestion. The same degradation pattern regardless of the presence or absence of CL (Fig. 4C) suggests that the hydrophobic residues are responsible for insertion into the membrane bilayer.

### Overproduction of PmtA results in formation of intracellular structures in *A. tumefaciens.*

**FIG 5:**
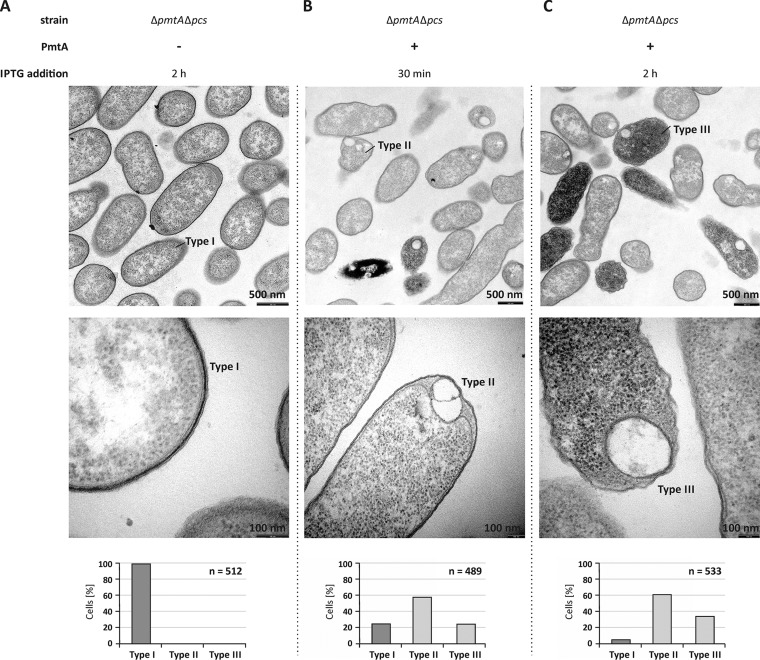
Intracellular structures are formed upon overproduction of PmtA in *A. tumefaciens*. (A to C) *A. tumefaciens* Δ*pmtA* Δ*pcs* cells carrying pTRC200 empty vector (A) or pTRC200_*pmtA* expression plasmid (B and C) were grown to the logarithmic growth phase (OD_600 _of 1.5). Protein production was induced using 100 µM IPTG. Cell morphology was analyzed by transmission electron microscopy 30 min and 2 h after the induction of PmtA overproduction. Bacterial cells were fixed, embedded, ultrathin sectioned, and analyzed using a Philips CM100 electron microscope. Images were taken with a CCD camera (Orius SC600; Gatan, Inc.). Three different cell types were observed. The bottom images show magnifications of selected cells, and the graphs below show quantification of the cell types (*n* is the total number of counted cells). Type I, initial morphology of *A. tumefaciens* Δ*pmtA* Δ*pcs* cells; type II, cells carrying vesicle-like structures; type III, cells with vesicle-like structures, high electron density, and damaged cell membrane.

Our *in vitro* results suggested a membrane-shaping activity of PmtA. To confirm this hypothesis, we analyzed thin sections of *A. tumefaciens* cells overexpressing *pmtA* via TEM. As shown in Fig. 5A, cells carrying the empty vector showed a regular cell shape (type I) with an intact inner and outer membrane identical to wild-type *A. tumefaciens* cells (data not shown). Strikingly, *pmtA* overexpression resulted in profound morphological changes (Fig. 5B and C). After 30 min of PmtA production, about 60% of the cells formed intracellular structures (type II) (Fig. 5B), and in addition to these hypothetical vesicle-like structures, 20% of the cells contained a higher electron density (type III) (Fig. 5B). The number of the type III cells increased with prolonged *pmtA* expression (Fig. 5C). The observed cellular changes were PmtA specific, since overproduction of green fluorescent protein (GFP) as a control did not affect cell morphology or electron density (Fig. S9).

10.1128/mBio.02082-16.9 FIG S9  Thin sections of *A. tumefaciens* cells expressing *gfp* analyzed via transmission electron microscopy (TEM). *A. tumefaciens* Δ*pmtA* Δ*pcs* cells carrying pTRC200 empty vector (left) or pTRC200_*gfp* expression plasmid (right) were grown until the logarithmic growth phase (OD_600_ of 1.5). Protein production was induced with 100 µM IPTG. Cell morphology was analyzed after 2 h of protein induction. Bacterial cells were fixed, embedded, ultrathin sectioned, and analyzed using a Philips CM100 electron microscope. Images were taken with a CCD camera (Orius SC600; Gatan, Inc.). One type of cells was observed, namely, cells that contain intact inner and outer membranes and no internal membrane structures (type I cells). In the graph, a quantification of the cell types is given (*n* is the total number of counted cells). Download FIG S9, JPG file, 0.3 MB. Copyright © 2017 Danne et al. 2017 Danne et al. This content is distributed under the terms of the Creative Commons Attribution 4.0 International license.

**FIG 6:**
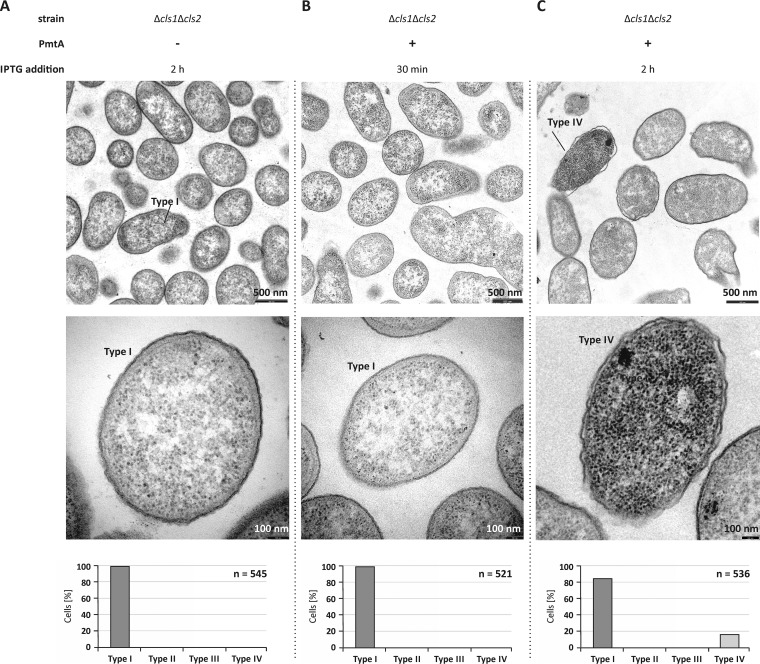
Intracellular structures are not formed in a CL-deficient mutant. (A to C) *A. tumefaciens* Δ*cls1* Δ*cls2* cells carrying pTRC200 empty vector (A) or pTRC200_*pmtA* expression plasmid (B and C) were observed via transmission electron microscopy. The experimental procedure and preparation of bacterial cells were performed as described in the legend to Fig. 5. The bottom images show magnifications of selected cells, and the graphs below show quantification of the cell types (*n* is the total number of counted cells). Two different types of cells were observed. Type I, cells with intact inner and outer membranes and no internal vesicles; type IV, cells with damaged membrane integrity and high electron density.

As CL is crucial for liposome deformation by PmtA (23), we investigated the formation of the intracellular structures in the CL-deficient *A. tumefaciens* Δ*cls1* Δ*cls2* strain (30). Almost all CL-deficient cells expressing *pmtA* were normally shaped similar to the control cells and did not contain intracellular vesicle-like structures (Fig. 6A to C), clearly demonstrating the importance of CL in the formation of the hypothetical vesicle-like structures by PmtA. After long *pmtA* expression, a small number of cells (20%) were damaged and contained a higher electron density (type IV) (Fig. 6C).

## DISCUSSION

The spatial organization and remodeling of bacterial membranes have become emerging areas of microbiological research. In this study, we provide the first evidence for membrane remodeling by a bacterial phospholipid *N*-methyltransferase, which synthesizes the methylated PE derivatives MMPE, DMPE, and PC in the plant pathogen *A. tumefaciens*. PmtA appears to be a bifunctional protein. Besides its function in PC formation, it has membrane-shaping activity *in vitro*.

### PmtA, a moonlighting protein with two distinct functions? 

This study revealed that the bacterial phospholipid-methylating enzyme PmtA has membrane deformation capacity and converts liposomes either into tubules or small vesicles. In addition to this *in vitro* membrane-remodeling activity of PmtA, our results suggest a membrane deformation activity *in vivo*. Upon overproduction of PmtA in *A. tumefaciens*, vesicle-like structures were observed in the cytoplasm via TEM. Formation of similar intracellular structures after expression of artificial fusion proteins in *E. coli* was recently reported by Huber et al. (39). The authors produced fusions containing GFP and amphiphilic domains from elastin-like proteins in *E. coli* and noticed the formation of membrane vesicles, which they designated *de novo* organelle-like structures. The production of membrane-bound organelles is well established in magnetotactic bacteria (20). These magnetosomes are cellular compartments and represent model systems for studying the biogenesis of bacterial organelles. Magnetosome formation is highly dynamic and requires the bacterial actin-like protein MamK (19, 40). Production of intracellular membrane vesicles was observed in *E. coli* after heterologous expression of caveolin from *Caenorhabditis elegans* and monotopic glucosyltransferase (MGS) from *Acholeplasma laidlawii* (41, 42). The intracellular structures formed in *A. tumefaciens* upon overproduction of PmtA might likewise represent intracellular vesicles. The observed structures seem to be enclosed by a lipid membrane. Although we consider it unlikely, at present, we cannot rule out the formation of inclusion bodies of overproduced PmtA, which might locally perturb the membrane structure. Therefore, we cannot unambiguously prove that the observed structures are intracellular vesicles. However, an interesting observation in support of this assumption was made by Kaneshiro and Law (43) in 1964. Here, PmtA was copurified with large amounts of microsome-like particles (43).

What might be the biological function of the membrane-shaping activity of PmtA? The observed strong membrane-shaping activity of PmtA could serve an important function in membrane structuring or maintenance *in vivo*. Other bacterial proteins such as the dynamin-like protein from *N. punctiforme* were shown to alter the morphology of vesicle structures *in vitro* and were shown to have a function in cell division (11, 44). In addition, MreB from *B. subtilis* and *E. coli* have been shown to induce local negative membrane curvature *in vitro* and seem to affect membrane fluidity, which is crucial for the interaction of several membrane proteins with the lipid bilayer (18, 45, 46). Likewise, local membrane curvature induction by PmtA might be required for membrane recruitment or activity of other membrane proteins in *A. tumefaciens*. Alternatively, the protein might rigidify specific membrane domains enriched in CL. Finally, local curvature induction by PmtA might be important for the enzyme itself and might be necessary for efficient catalysis of the three consecutive methylation reactions.

**FIG 7:**
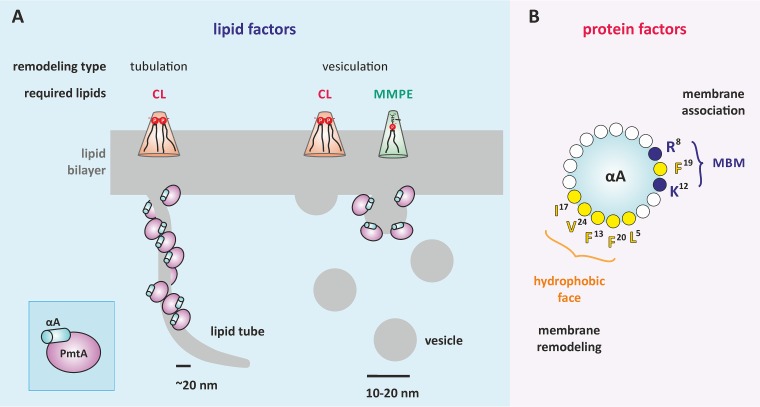
Requirements for liposome-shaping activity of PmtA**.** (A) The dual liposome-remodeling capacity of PmtA is dependent on the membrane lipid composition. Membrane tubulation is promoted by the presence of cardiolipin (CL), whereas vesiculation of membranes requires CL and monomethyl-phosphatidylethanolamine (MMPE). Proteins, membrane tubules, and vesicles are not drawn to scale. (B) The N-terminal lipid-binding helix αA is important for membrane deformation by PmtA. αA is a multifunctional domain involved in membrane adsorption by the membrane-binding motif (MBM) comprised of R8, K12, and F19 (23) and membrane shaping by the predicted hydrophobic face.

Membrane remodeling by PmtA strictly depends on the lipid composition. Figure 7A summarizes which lipids influence the liposome-shaping activity of PmtA. Both membrane tubulation and vesiculation require the presence of CL. Consistent with this finding, the formation of vesicle-like structures upon overexpression of PmtA is abrogated in a CL-deficient mutant strain, supporting the idea that CL is important for the membrane-shaping activity of PmtA *in vivo*. Similar to PmtA, liposome deformation by the eukaryotic membrane fission protein BARS (brefeldin A ADP-ribosylated substrate) is lipid specific and requires the presence of PA (47). Interestingly, it was reported recently that the bacterial membrane-remodeling protein FisB from *B. subtilis* exploits interaction with CL for membrane fusion and lipid mixing *in vitro* (14), suggesting a common CL-dependent membrane-shaping process of bacterial membrane-deforming proteins. However, the precise mechanisms underlying membrane deformation by bacterial proteins are unknown. Limited proteolysis experiments suggest an insertion of the αA region of PmtA into the core of the lipid bilayer depending on the presence of CL. This might induce a conformational change necessary for the membrane-remodeling activity of PmtA. According to protein modeling, a common feature of bacterial proteins forming intracellular membrane vesicles (IMVs) is a Rossmann-fold tertiary structure (16, 24, 48), suggesting a correlation between the tertiary structure of proteins and membrane deformation.

Phospholipids other than CL influence membrane deformation by PmtA. The products of liposome remodeling by PmtA (tubules or vesicles) depend on the methylation intermediates, namely, MMPE and with less efficiency, DMPE. Both are zwitterionic lipids with intrinsic conical shape. Binding of PmtA to model membranes containing PE, PG, and CL led to preferential formation of cylindrical membrane tubules, whereas the addition of MMPE to the liposomes promotes vesiculation (Fig. 7A). These results indicate that the outcome of dual membrane remodeling by PmtA depends on the local amount of CL, MMPE, and DMPE in the membrane. Based on the characterization of eukaryotic curvature-inducing proteins like BAR (Bin-amphiphysin-Rvs) domain proteins that are required for membrane dynamics (49), it is proposed that tubulation or vesiculation of membranes relies on two different mechanisms for interaction of these proteins with the lipid bilayer (50). Crescent-like protein scaffolds deform the membrane into cylindrical membrane tubes and are not proposed to generate membrane fission. In contrast, shallow hydrophobic insertions drive membrane fission and result in the transformation of membranes into separated small vesicles. Differential membrane shaping by PmtA might likewise rely on such interactions with the membrane (Fig. 7A).

### αA is decisive for membrane remodeling by PmtA.

The N-terminal αA helix is a specialized protein module that, apart from its role in membrane recruitment and enzyme activity (23), is equally important for shaping or remodeling the membrane, similar to eukaryotic BAR domains (51). Similar lipid-binding helices are known to be involved in membrane-remodeling machineries in eukaryotes (52). The eukaryotic CTP:phosphocholine cytidylyltransferase (CCT) catalyzing the rate-limiting step in PC formation contains a membrane-induced amphipathic helix with curvature-sensing features (53, 54). Several membrane-sculpting proteins bend the membrane by interaction of hydrophobic residues with the lipid bilayer as demonstrated for alMGS synthase from *A. laidlawii* or eukaryotic Pex11p (16, 55). Accordingly, the hydrophobic residues (F20 and V24) in αA, which are oriented on the opposite site of the membrane-binding motif (Fig. 7B), are crucial for membrane remodeling by PmtA. Moreover, trypsin protection assays suggest insertion of those residues into the core of the lipid bilayer. We propose that αA is a multifunctional domain of PmtA required for membrane adsorption and enzyme activity (23) and also for membrane remodeling. This is achieved by two opposing faces of the helix (Fig. 7B).

## MATERIALS AND METHODS

### Materials.

1,2-Dioleoyl-*sn*-glycero-3-phosphoethanolamine-*N*-methyl, 1,2-dioleoyl-*sn*-glycero-3-phosphoethanolamine-*N*,*N*-dimethyl, 1,2-dioleoyl-*sn*-glycero-3-phospho-(1′-*myo*-inositol), 1,2-dioleoyl-*sn*-glycero-3-phospho-(1′-*myo*-inositol-4′-phosphate), 1′,3′-bis(1,2-dioleoyl-*sn*-glycero-3-phospho)-*sn*-glycerol, and 1,2-dioleoyl-*sn*-glycero-3-phosphate were purchased from Avanti Polar Lipids, Inc. l-α-Phosphatidylethanolamine (dioleoyl), cardiolipin from bovine heart (C_18:2_), and 1,2-dioleoyl-*sn*-glycero-3-phospho-*rac*-(1-glycerol) were obtained from Sigma-Aldrich. Uranyl acetate, glycid ether 100, and trypsin (from porcine pancreas; 60 U/mg) were from Serva.

### Bacterial strains and growth conditions.

Bacterial strains used in this study are listed in Table S1A in the supplemental material. *A. tumefaciens* cells were cultivated in Luria-Bertani (LB) medium supplemented with 100 μg ml^−1^ spectinomycin at 30°C. *E. coli* cells were grown in LB medium supplemented with 50 μg ml^−1^ kanamycin at 37°C. *E. coli* JM83 was used for cloning, and *E. coli* BL21(DE3) was used for heterologous expression of recombinant proteins.

10.1128/mBio.02082-16.10 TABLE S1 (A) Bacterial strains and plasmids used in this study. (B) Oligonucleotides used in this study. Download TABLE S1, DOCX file, 0.02 MB. Copyright © 2017 Danne et al. 2017 Danne et al. This content is distributed under the terms of the Creative Commons Attribution 4.0 International license.

### Preparation of thin sections of *A. tumefaciens* and *E. coli* cells for transmission electron microscopy.

Thin sections of *A. tumefaciens* and *E. coli* cells were prepared by the method of Wenzel et al. (56) with some modifications. Briefly, *A. tumefaciens* and *E. coli* cells carrying appropriate expression plasmids were grown as described above until the logarithmic growth phase (optical density at 600 or 580 nm [OD_600/580_] of ~1.6). Protein production was induced with 100 µM isopropyl-β-d-thiogalactopyranoside (IPTG). After the indicated time points, cells were harvested and incubated with fixative solution containing 2% glutaraldehyde for 20 min. The cells were washed twice with double-distilled water and incubated with 2% uranyl acetate for 5 min. The samples were washed twice with double-distilled water and stained with 2% osmium tetroxide. The cells were harvested and dehydrated by incubation in a series of solutions with 50%, 70%, 90%, and 100% acetone for 5 min each. The cells were incubated with 1:1 acetone-epoxy resin for 15 min and afterward embedded in pure epoxy resin. For preparation of the epoxy resin, solution A (38% glycid ether 100–62% dodecyl succinic anhydride) was mixed with solution B (47% glycid ether 100–53% methylnadic anhydride) at a ratio of 3:7. To this mixture, tris-2,3,6-(dimethylaminomethyl)phenol (DMP30) was added to a final concentration of 1.5% to accelerate polymerization. Polymerization was performed at 75°C for 18 h. The polymerized blocks were cut into a series of slices of 50-nm thickness. Ribbons of sections were transferred on Formvar-coated copper grids. Sections were observed via a Philips CM100 electron microscope. The images were taken with a charge-coupled-device (CCD) camera (Orius SC600; Gatan, Inc.) and acquired with Gatan software.

### Cloning and site-directed mutagenesis.

To construct plasmid pBO1234, the *pmtA* coding region was cloned from plasmid pBO832 into the broad-host-range expression vector pTRC200 via NcoI and SalI restriction sites. Site-directed mutagenesis was performed using a QuikChange mutagenesis kit (Stratagene) according to the manufacturer’s protocol, and oligonucleotides are listed in Table S1B. Plasmid pBO832 was used as the template and subjected to site-directed mutagenesis to create PmtA variants. The resulting plasmids were verified via DNA sequencing (MWG Eurofins).

### Expression and purification of recombinant His-tagged proteins in *E. coli.*

Recombinant proteins were expressed in *E. coli* and purified as described previously (23).

### Liposome preparation.

For liposome preparation, we used artificial lipids or extracted lipids from different *A. tumefaciens* strains. All lipids obtained from commercial sources contained C_18:1_ fatty acids. Exceptions are indicated in the figures. To prepare liposomes with *A. tumefaciens* lipids, cells were cultivated as described above to an optical density of OD_600_ of ~3.0. Equal amounts of cells were harvested by centrifugation and washed with A. dest. Lipids were extracted by the method of Bligh and Dyer (58) and dried in glass tubes under nitrogen flow. Liposomes were prepared as described previously (22) using an Avanti miniextruder according to the manufacturer’s instructions. Polycarbonate membranes with different pore sizes were used to prepare appropriate sized liposomes. The experiments were performed with 400-nm liposomes; exceptions are mentioned in the figures.

When commercial lipids were used, appropriate amounts of lipids in solutions with chloroform as a solvent were dried under nitrogen flow in glass tubes, and liposomes were prepared as described in reference 22. Lipid composition and concentrations are indicated in the figures.

### Liposome cosedimentation assay.

Liposome cosedimentation assays were performed as described previously (23). Samples contained 10 µM concentration of recombinant protein and 0.75 mM liposomes. Lipid compositions are indicated in the figures. Results of liposome cosedimentation assays were analyzed by SDS-PAGE and Western blot analysis. His-tagged proteins were detected using a penta-His-horseradish peroxidase (HRP) conjugate (Qiagen). Chemiluminescent bands were visualized on ECL Hyperfilms (Amersham) by the use of ECL Western blotting detection reagent (GE Healthcare).

### Liposome-remodeling assay.

Recombinant PmtA (10 µM) was incubated with liposomes for 30 min at room temperature in KH_2_PO_4_ buffer (50 mM) (pH 8.0) (exceptions are mentioned in Results or figure legends). The compositions of the liposomes used are indicated in the figures. Liposomes were spotted on Formvar-coated copper grids; buffer was removed after 5 min using Whatman paper. Liposomes were negatively stained with 1% uranyl acetate. Preparations were analyzed via a Philips 420 transmission electron microscope equipped with a Gatan digital camera. We used 10-nm gold particles to estimate the size of liposomes or liposomal structures for the appropriate magnification.

### Protease protection assay.

Trypsin protection assay was performed in the presence and absence of liposomes. Recombinant PmtA (10 μM) was incubated with or without liposomes (0.75 mM; protein/lipid ratio 1:75) at room temperature for 30 min in KH_2_PO_4_ buffer (50 mM; pH 8.0). The compositions of the liposomes used are indicated in the figures. After incubation, trypsin was added to the samples at a final concentration of 10 µg ml^−1^. Digestion of PmtA was analyzed via SDS-PAGE after different time points (0, 2, 5, 7, 15, and 30 min). Samples were supplemented with SDS sample buffer and boiled to inactivate the protease. SDS sample buffer contained 1 M Tris (pH 6.8), 50% glycerol (vol/vol), 10% SDS (wt/vol), 0.5% bromophenol blue (wt/vol), and 5% β-mercaptoethanol (vol/vol).

### Secondary structure analysis via CD spectroscopy.

The secondary structures of recombinant PmtA variants were analyzed via circular dichroism (CD) spectroscopy. CD spectra of recombinant proteins were recorded 10 times between 200 and 270 nm in 50 mM potassium buffer pH 8.0 with a Jasco 715 spectropolarimeter at room temperature. The samples contained 5 μM concentration of recombinant proteins in a total volume of 200 µl.
