# Supplementary figures and images for: Membrane Remodeling by a Bacterial Phospholipid-Methylating Enzyme
Source: mBio. 2017 Feb 14;8(1):e02082-16. doi: 10.1128/mBio.02082-16 (PMC5312082; doi:10.1128/mBio.02082-16)

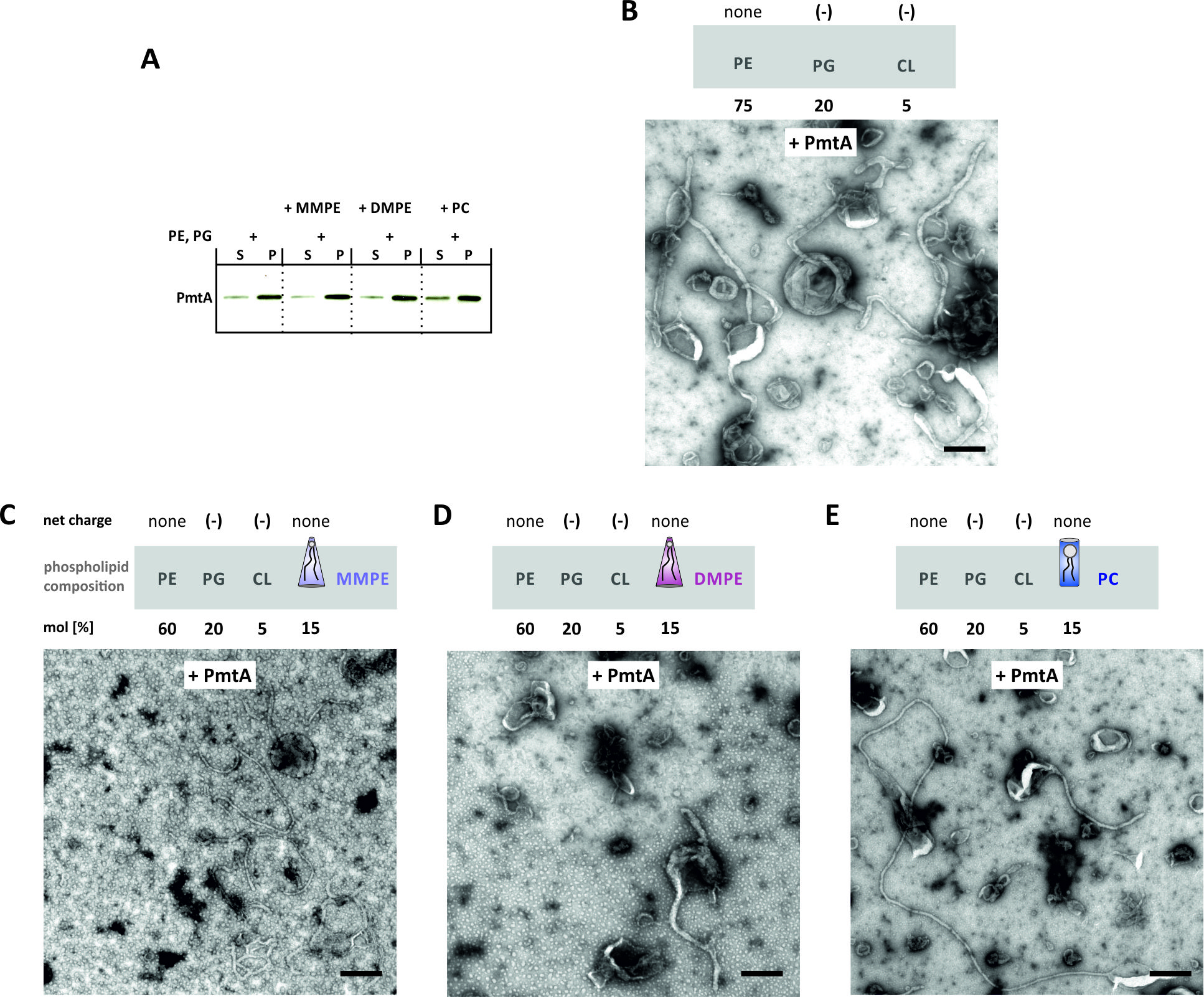

Supplement: FIG S1 [file mbo001173193sf1.jpg]

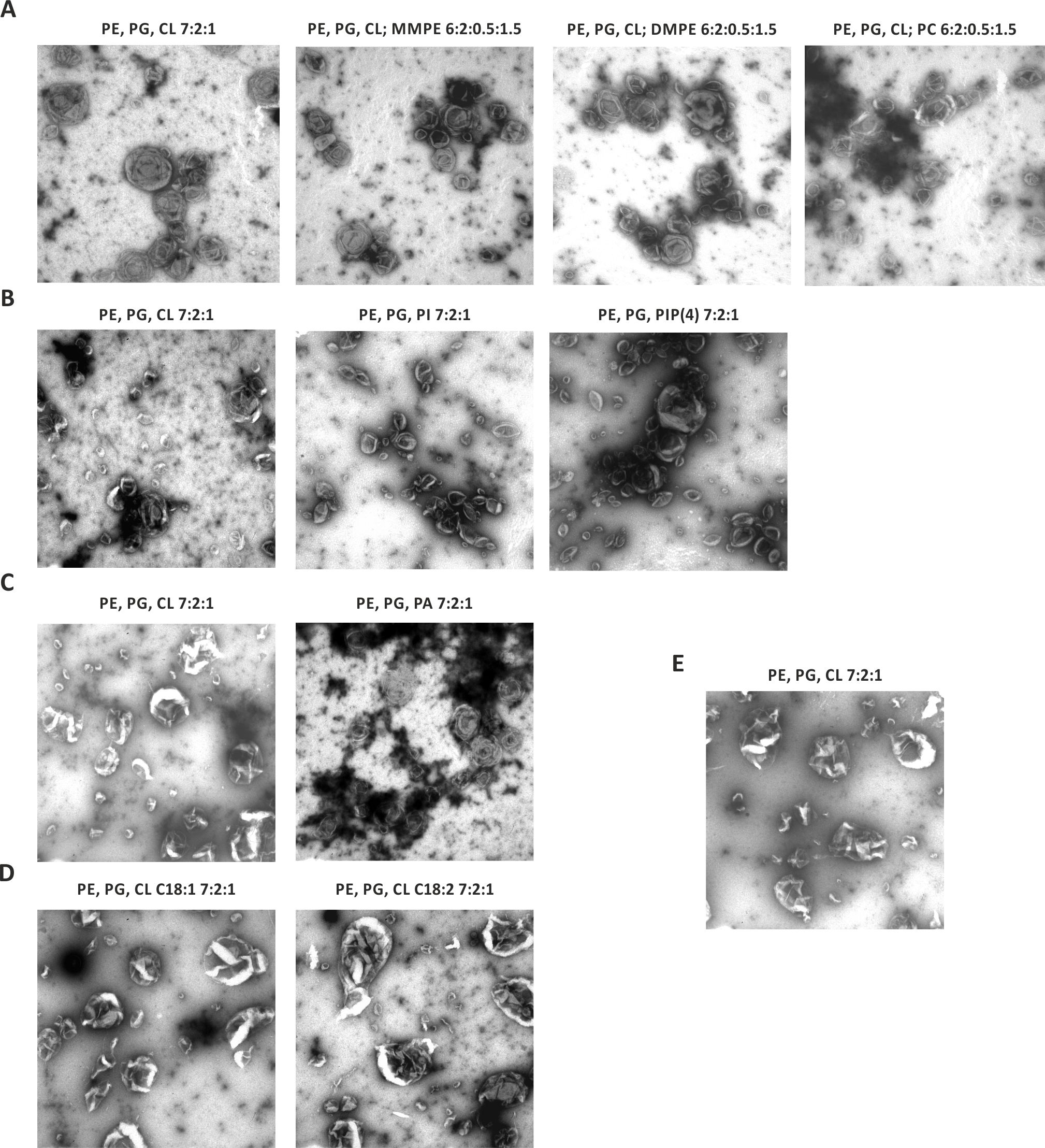

Supplement: FIG S2 [file mbo001173193sf2.jpg]

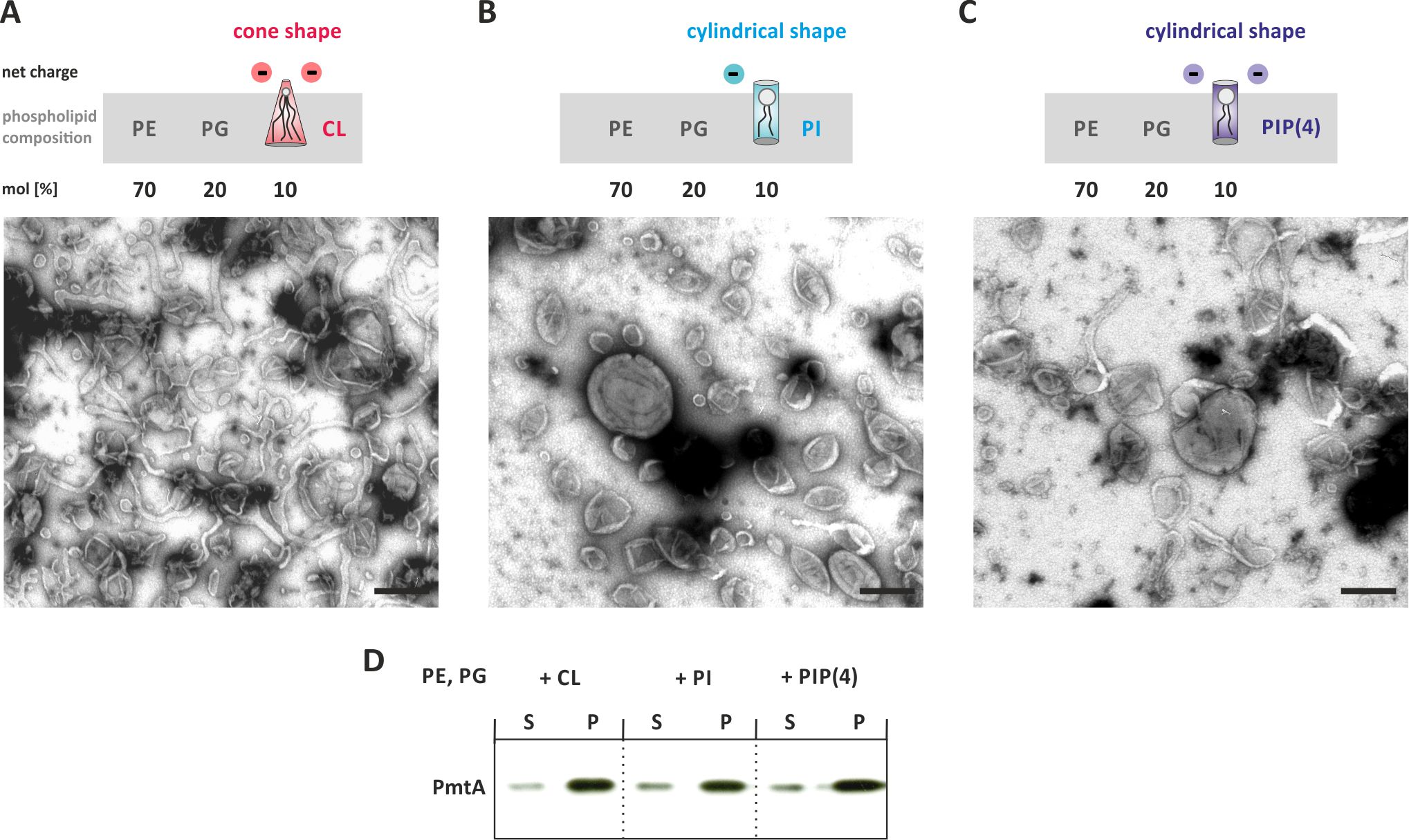

Supplement: FIG S3 [file mbo001173193sf3.jpg]

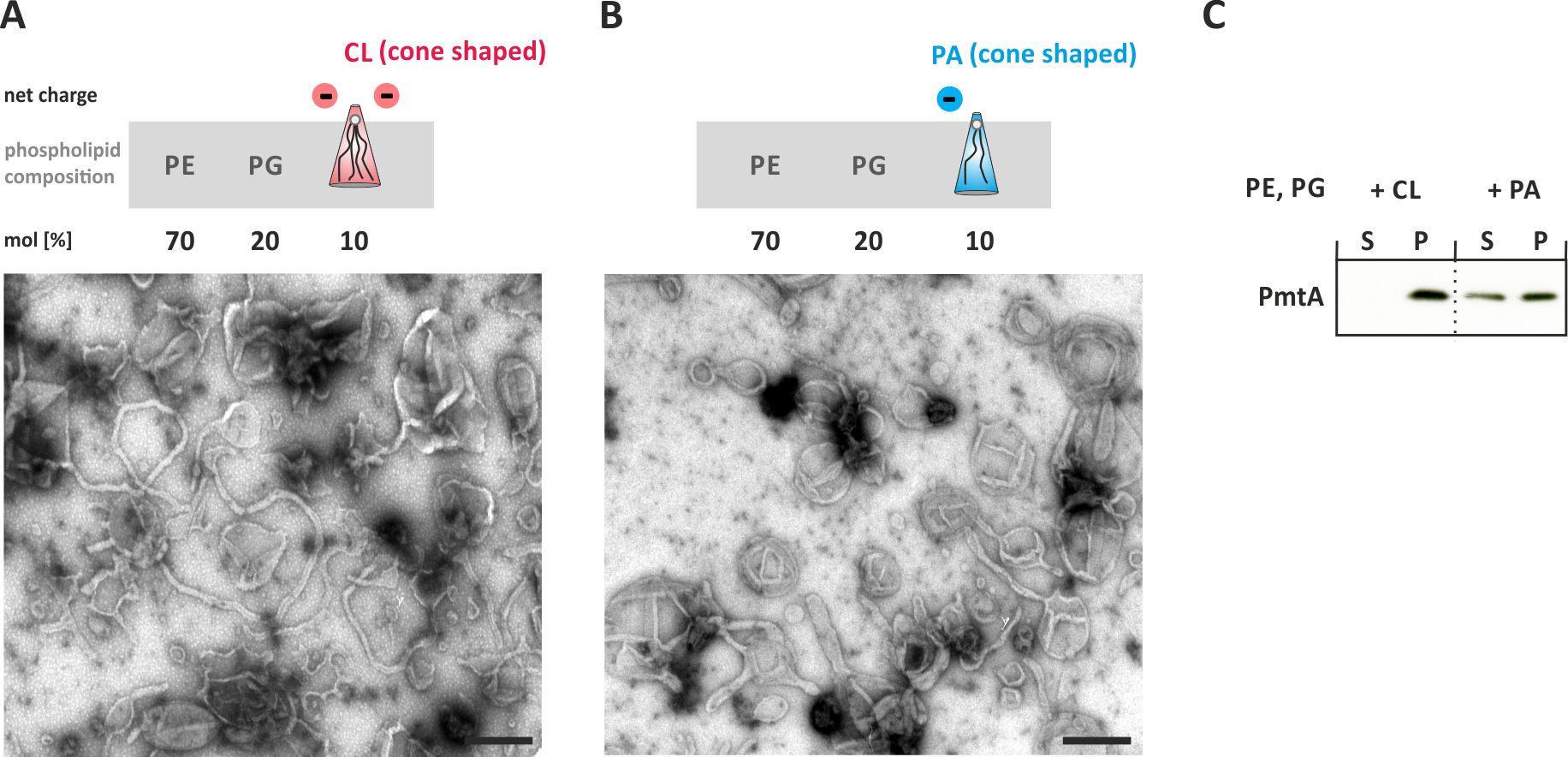

Supplement: FIG S4 [file mbo001173193sf4.jpg]

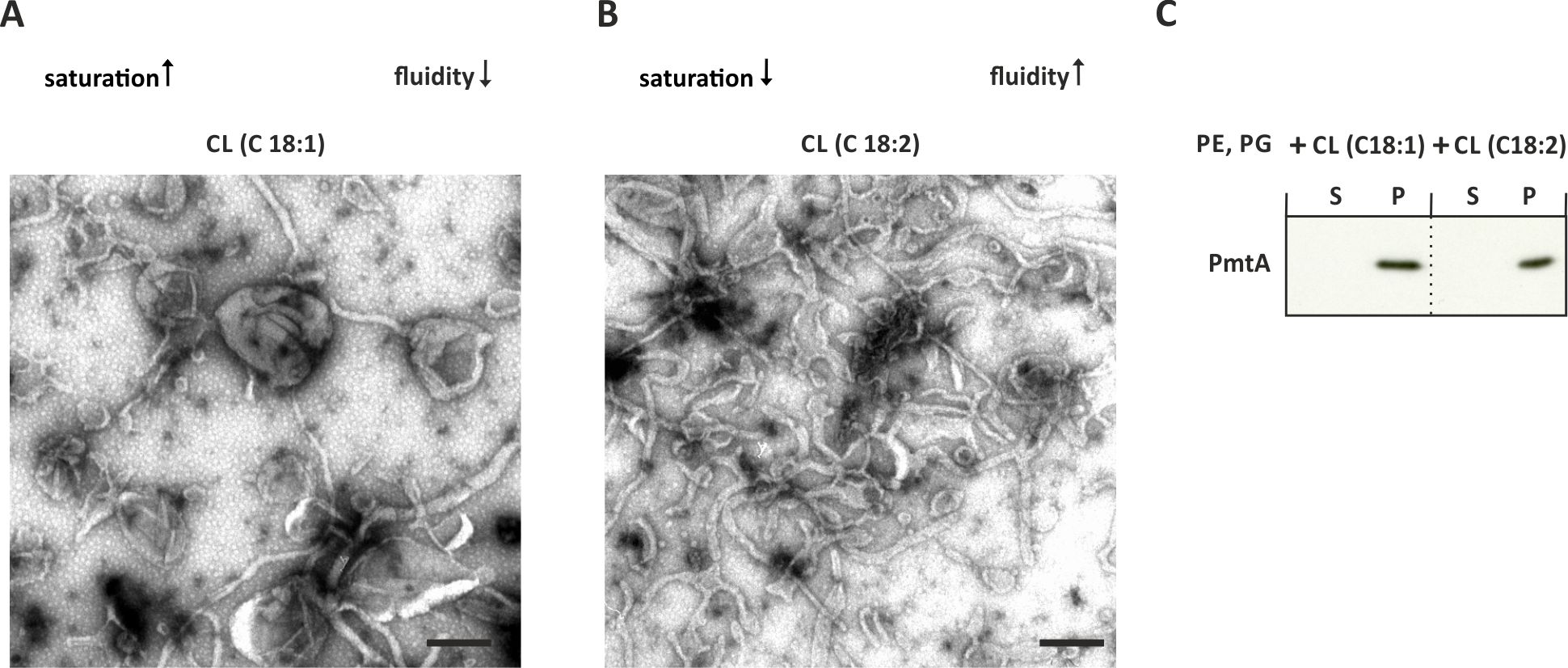

Supplement: FIG S5 [file mbo001173193sf5.jpg]

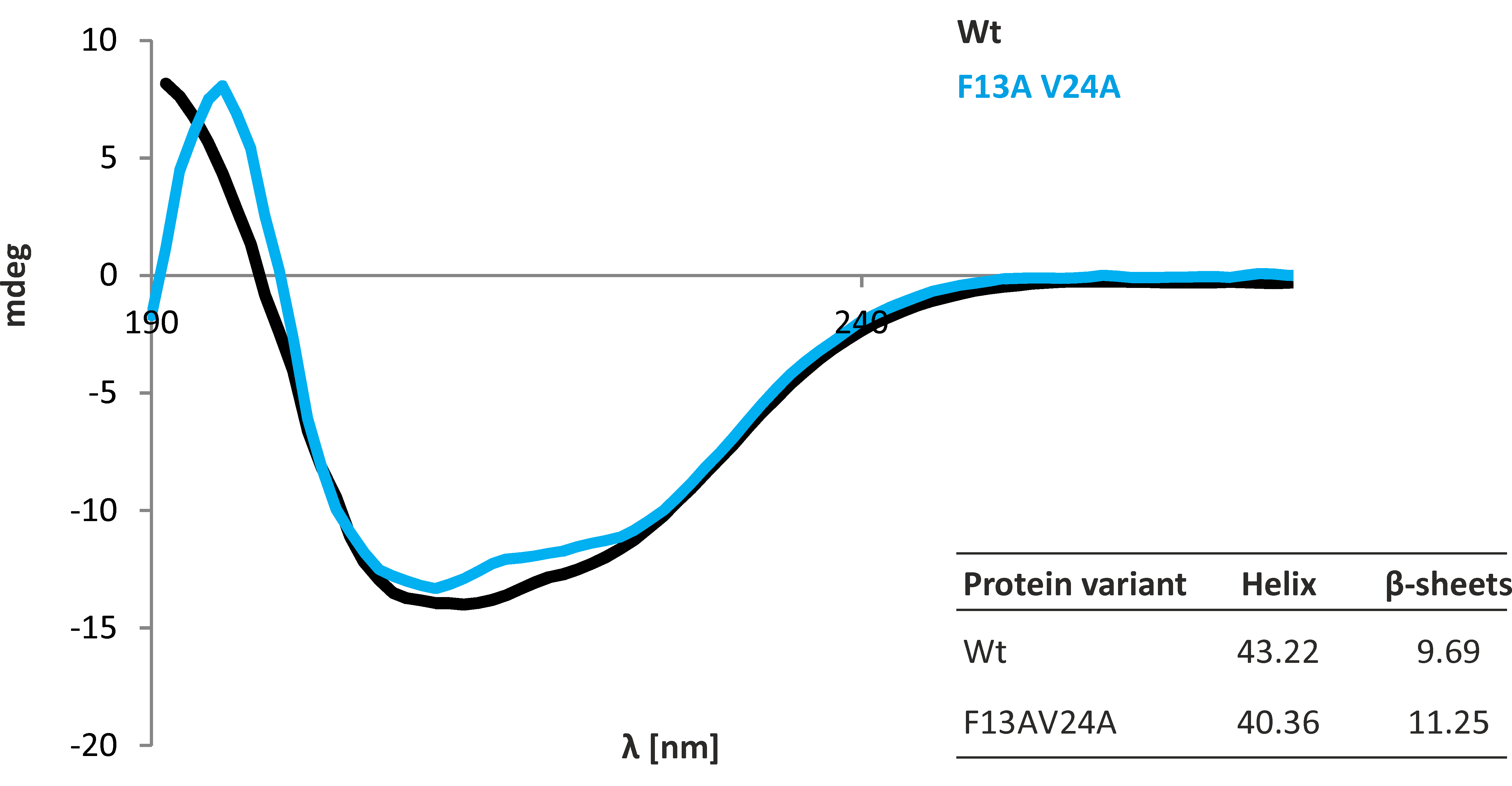

Supplement: FIG S6 [file mbo001173193sf6.tif]

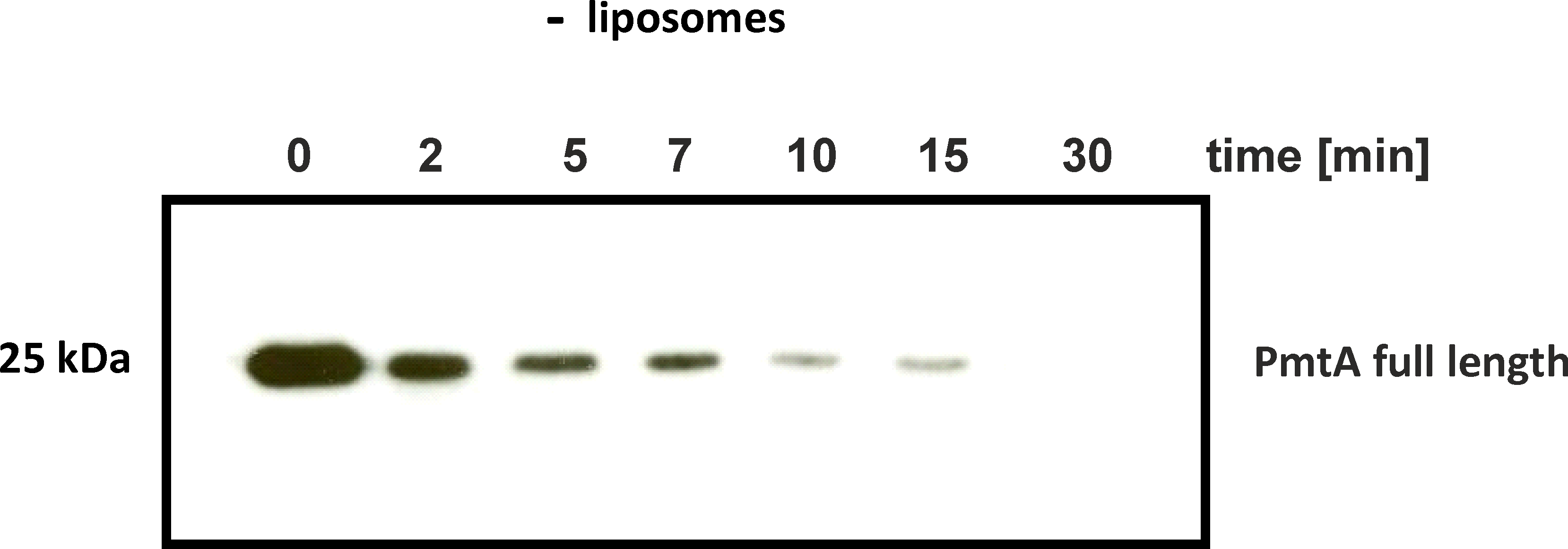

Supplement: FIG S7 [file mbo001173193sf7.tif]

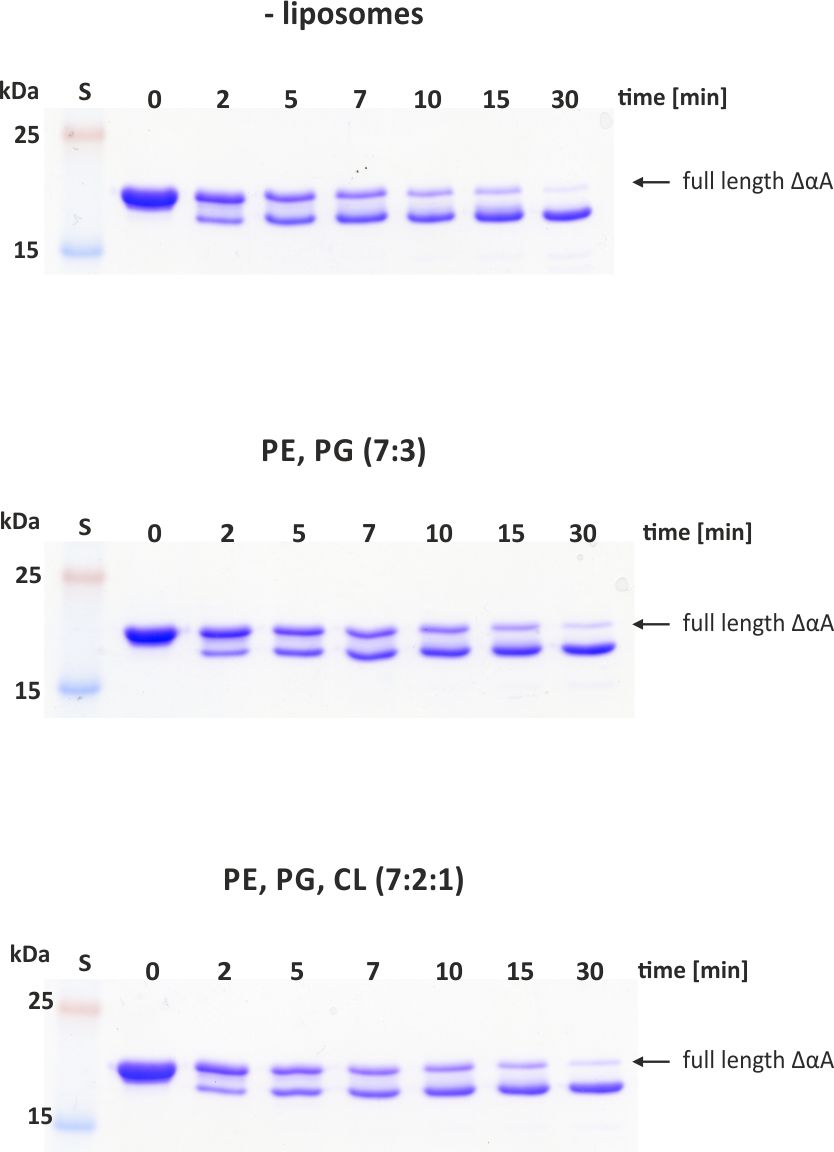

Supplement: FIG S8 [file mbo001173193sf8.jpg]

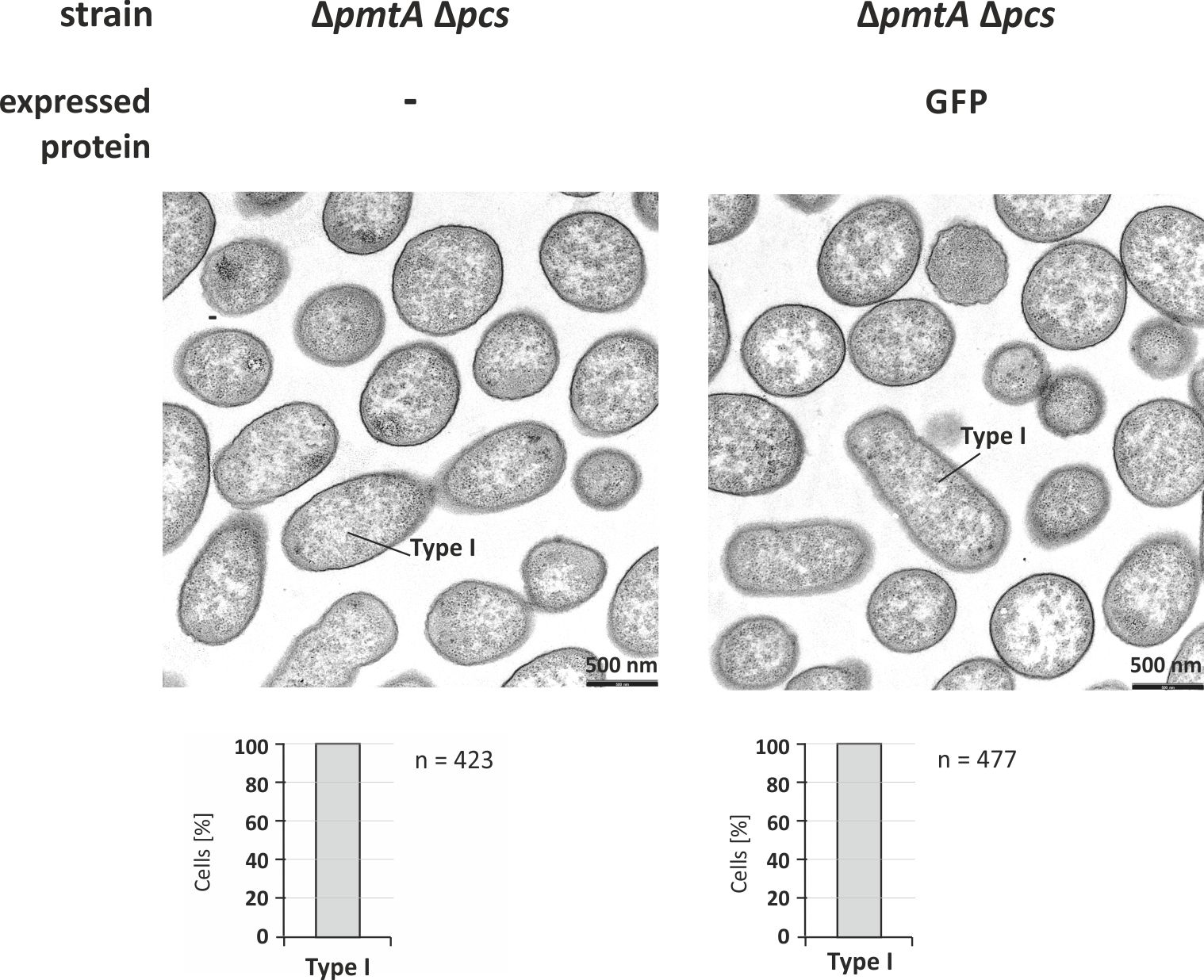

Supplement: FIG S9 [file mbo001173193sf9.jpg]
