# Supplementary material for: Membrane Remodeling by a Bacterial Phospholipid-Methylating Enzyme
Source: mBio. 2017 Feb 14;8(1):e02082-16. doi: 10.1128/mBio.02082-16 (PMC5312082; doi:10.1128/mBio.02082-16)
Supplement: TABLE S1 [file mbo001173193st1.docx]

**Table S1A Bacterial strains and plasmids used in this study**

| **Strain/ plasmid** | **Characteristics** | **Reference** |
| --- | --- | --- |
| ***Escherichia coli* strains** |  |  |
| E. coli JM83 | Cloning host | ([1](#_ENREF_1)) |
| E. coli Bl21 DE3 | Host for expression of recombinant proteins | ([2](#_ENREF_2)) |
| ***Agrobacterium tumefaciens strains*** |  |  |
| A. tumefaciens C58 | Wild type | C. Baron |
| C58 ΔpmtA | Wild-type derivative, deletion of the pmtA gene | ([3](#_ENREF_3)) |
| C58 Δpcs | Wild-type derivative, deletion of the pcs gene | ([3](#_ENREF_3)) |
| C58 ΔpmtAΔpcs | Wild-type derivative, deletion of the pmtA and pcs genes | ([3](#_ENREF_3)) |
| C58 Δ*cls*1Δ*cls*2 | Wild-type derivative, deletion of the cls1 and cls2 genes | ([4](#_ENREF_4)) |
| **Plasmid** |  |  |
| pBO832 | Wt-PmtA, pET28b | ([5](#_ENREF_5)) |
| pBO3776 | F19A-PmtA, pET28b | ([6](#_ENREF_6)) |
| pBO3773 | K6QR8QK12Q-Pmt, pET28b | ([6](#_ENREF_6)) |
| pBO3790 | F13AV24A-PmtA, pET28b | this study |
| pBO3751 | ΔαA-PmtA, pET28b | ([6](#_ENREF_6)) |
| pBO1234 | Wt-PmtA, pTRC200 | this study |

**Table S1B Oligonucleotides used in this study**

| **Oligonucleotide** | **5′-3′** (base-pair exchanges are underlined) |
| --- | --- |
| F13AV24A_1 | AGCTGAGGAAGAAATCCGCTTTTTCAAAGGTATGGCCAG |
| F13AV24A_2 | CTGGCCATACCTTTGAAAAAGCGGATTTCTTCCTCAGCT |

***References***

1. **Yanisch-Perron C, Vieira J, Messing J.** 1985. Improved M13 phage cloning vectors and host strains: nucleotide sequences of the M13mp18 and pUC19 vectors. Gene **33:**103-119.

2. **Studier FW, Moffatt BA.** 1986. Use of Bacteriophage-T7 Rna-Polymerase to Direct Selective High-Level Expression of Cloned Genes. J of Mol Biol **189:**113-130.

3. **Wessel M, Klüsener S, Gödeke J, Fritz C, Hacker S, Narberhaus F.** 2006. Virulence of *Agrobacterium tumefaciens* requires phosphatidylcholine in the bacterial membrane. Mol Microbiol **62:**906-915.

4. **Czolkoss S, Fritz C, Hölzl G, Aktas M.** 2016. Two distinct cardiolipin synthases operate in *Agrobacterium tumefaciens*. PLoS One **11:**e0160373.

5. **Aktas M, Narberhaus F.** 2009. *In vitro* characterization of the enzyme properties of the phospholipid *N*-methyltransferase PmtA from *Agrobacterium tumefaciens*. J Bacteriol **191:**2033-2041.

6. **Danne L, Aktas M, Gleichenhagen J, Grund N, Wagner D, Schwalbe H, Hoffknecht B, Metzler-Nolte N, Narberhaus F.** 2015. Membrane-binding mechanism of a bacterial phospholipid *N*-methyltransferase. Mol Microbiol **95:**313-331.
